# Systems Biology Analysis of Temporal Dynamics That Govern Endothelial Response to Cyclic Stretch

**DOI:** 10.3390/biom12121837

**Published:** 2022-12-08

**Authors:** Michael W. Lai, Nathan Chow, Antonio Checco, Balvir Kunar, David Redmond, Shahin Rafii, Sina Y. Rabbany

**Affiliations:** 1 Bioengineering Program, DeMatteis School of Engineering and Applied Science, Hofstra University, New York, NY 11549, USA; 2 Division of Regenerative Medicine, Ansary Stem Cell Institute, Weill Cornell Medicine (WCM), New York, NY 10065, USA

**Keywords:** endothelial cells, mechanotransduction, RNA-seq, vascular biology

## Abstract

Endothelial cells in vivo are subjected to a wide array of mechanical stimuli, such as cyclic stretch. Notably, a 10% stretch is associated with an atheroprotective endothelial phenotype, while a 20% stretch is associated with an atheroprone endothelial phenotype. Here, a systems biology-based approach is used to present a comprehensive overview of the functional responses and molecular regulatory networks that characterize the transition from an atheroprotective to an atheroprone phenotype in response to cyclic stretch. Using primary human umbilical vein endothelial cells (HUVECs), we determined the role of the equibiaxial cyclic stretch in vitro, with changes to the radius of the magnitudes of 10% and 20%, which are representative of physiological and pathological strain, respectively. Following the transcriptome analysis of next-generation sequencing data, we identified four key endothelial responses to pathological cyclic stretch: cell cycle regulation, inflammatory response, fatty acid metabolism, and mTOR signaling, driven by a regulatory network of eight transcription factors. Our study highlights the dynamic regulation of several key stretch-sensitive endothelial functions relevant to the induction of an atheroprone versus an atheroprotective phenotype and lays the foundation for further investigation into the mechanisms governing vascular pathology. This study has significant implications for the development of treatment modalities for vascular disease.

## 1. Introduction

Vascular endothelial cells (ECs) line the luminal surfaces of blood vessels and experience dynamic mechanical forces from their microenvironment. These stimuli result from the hemodynamic parameters exerted by blood flow and pressure and play a role in regulating the gene expression and physiological responses of ECs—known as mechanotransduction. Two important concurrent hemodynamic forces are shear stress and cyclic stretch. Shear stress is a result of the friction that is generated between blood flow and the endothelium, while cyclic stretch arises due to the pulsatile nature of blood flow [1]. Subsequent morphological changes and atheroprone or atheroprotective EC phenotypes result in a response to these stimuli.

The differentiation of atheroprone versus atheroprotective markers and morphologies is necessary for the investigation of the fine balance that ECs exhibit within the body, where subtle hemodynamic changes over the course of years can precipitate adverse health outcomes if not kept within ideal parameters. A wide range of sequelae from aberrant EC stress remain significant health concerns, such as atherosclerosis, thrombus formation, aneurysm, aortic dissection, and nephropathy. In the context of thrombi, for example, decreased EC vitality leads to diminished barrier function and the exposure of sub-endothelial collagen to platelets, leading to aggregation and vessel occlusion [2].However, the most prominent of these concerns is atherosclerosis, due to its driving role in vascular disease, which despite advances remains the number one cause of death in the US [3].

Both laminar shear stress and physiological cyclic stretch have been demonstrated to be essential in the maintenance of vascular homeostasis and are thus associated with an atheroprotective phenotype [4]. Meanwhile, disturbed blood flow imparts oscillatory shear stress and pathological cyclic stretch to the endothelium, disrupting appropriate EC mechanotransduction, and thus is associated with an atheroprone phenotype [5,6,7]. The mechanisms by which laminar and oscillatory shear stress induce an atheroprotective and atheroprone phenotype, respectively, have been well characterized [8,9,10]. For cyclic stretch, several alterations to the cytoskeleton and downstream cellular signaling have been reported [11]. These alterations occur due to signal transduction by the mechanosensitive receptors associated with ECs, including ion channels known as the transient receptor potential (TRP), which have a demonstrated role in stretch-mediated calcium influx, integrins, and the platelet endothelial cell adhesion molecule (PECAM-1) [12,13,14,15]. Transduction by these mechanoreceptors induces several hallmarks of the endothelial response to cyclic stretch, including actin stress fiber formation regulated by the small GTPase Rho and stretch-mediated angiogenesis [16,17]. The endothelial atheroprone phenotype brought about by pathological cyclic stretch disrupts vascular homeostasis through the increased production of reactive oxygen species (ROS) and increased inflammation [18,19]. However, the molecular events governing the progression from an atheroprotective to an atheroprone phenotype in response to cyclic stretch require further investigation. To better understand how the magnitude of the cyclic stretch affects cellular responses, we examined the level of mechanical stimuli in a quantitative manner to discern the relative contribution of different regulatory genes.

Identifying markers signifying EC vitality is a prerequisite for the analysis of the factors that influence it and lead to vascular disease. EC vitality can be monitored by EC proliferation, stiffness, inflammatory response, mTOR expression, and fatty acid metabolism. Proliferation ensures viable endothelium and barrier function. Stiffness refers to the EC’s ability to absorb hemodynamic stress, with excessive stiffness being a function of poor resilience. The EC modulation of inflammatory responses protects or promotes immune deposition-forming plaques, while fatty acid metabolism influences lipid deposition. Molecular Target of Rapamycin (mTOR) is linked with vasodilation, inflammation, cell survival, and cell proliferation and provides a comprehensive view of EC function. Therefore, each of these variables is useful in the study of EC vitality or the lack thereof [20].

The physiological and pathological responses of ECs to cyclic stretch have been primarily elucidated using experimental models that impose uniaxial cyclic stretch in vitro at a set magnitude [21]. For more physiological relevance, it is necessary for equibiaxial cyclic stretch to be applied to ensure greater strain homogeneity [22,23]. In this study, primary human umbilical vein endothelial cells (HUVECs) were subject to equibiaxial cyclic stretch at magnitudes in the range of 5% ∆r to 25% ∆r. Stretch magnitudes of 10% ∆r and 20% ∆r were utilized for the final RNA-sequencing (RNA- seq) analyses, which are often used in vitro to represent physiological and pathological conditions [24]. HUVECs were chosen for our experimental system given that they have been used previously to model cardiac pathologies due to their in vitro durability [8]. Smooth muscle cells are prone to differentiation when cultured in vitro, in the absence of natural stimuli such as high shear and pressure [25]. Once the HUVECs are cultured, these cells assume the molecular and cellular features of “generic endothelial cells”. We then performed a series of post-sequencing analyses to determine the molecular networks that drive the key endothelial responses to cyclic stretch (cell cycle regulation, inflammatory response, fatty acid metabolism, and mTOR signaling). Here, we utilize a systems biology-based approach to present a detailed mapping of the molecular networks which govern the dynamic transition from an atheroprotective to an atheroprone phenotype in response to cyclic stretch.

## 2. Materials and Methods

### 2.1. Endothelial Cell Culture

Primary HUVECs freshly isolated from two different vascular beds were cultured in Medium 199 (Corning, Corning, NY, USA), supplemented with l-glutamine (Corning, Corning, NY, USA), antibiotic antimycotic (Corning, Corning NY, USA), HEPES buffer (Corning, Corning, NY, USA), heparin (Sigma Aldrich, St. Louis, MO, USA), and 20% fetal bovine serum (Hyclone, Logan, UT, USA). The HUVECs were transfected with the E4ORF1 of the Ad E4 gene complex, which has previously been demonstrated to support the long-term survival of ECs through sustained Akt phosphorylation [26]. The cells were maintained within a trigas humidified Heracell 150i incubator (Thermo Scientific, Waltham, MA, USA) at 37 °C and 5% CO_2_/O_2_. For experiments to inhibit the mTOR’s kinase activity, the cells were pre-treated with rapamycin (Cell Signaling Technology, Danvers, MA, USA) at a concentration of 100 nM for 24 h prior to stretch.

### 2.2. Cyclic Stretch

A CellScale MCB1 (Cell Scale Biomaterials Testing, Ontario, Canada) device was adapted to deliver the equibiaxial cyclic stretch to the HUVECs seeded onto custom-built circular polydimethylsiloxane (PDMS) molds. To allow for the application of the equibiaxial stretch, the HUVECs were cultured on circular molds which were mounted within the stretch chamber of the device. The molds were cast using a Sylgard 184 Silicone Elastomer Kit (Dow Chemical, Midland, MI, USA) at a standard 10:1 ratio of base to curing agent. The PDMS molds were then cured for 24 h and sterilized under ultraviolet light. To assist the adhesion of the HUVECs to the PDMS, the molds were plasma treated for one minute using a PE-25 plasma cleaner (Plasma Etch, Carson City, NV, USA) and coated with fibronectin (Thermo Fisher Scientific, Waltham, MA, USA) at a concentration of 20 µg/mL. After 12 h of culture, the equibiaxial cyclic stretch was applied to the cells at physiologically relevant (10% change of radius (∆r)) and pathologically relevant (20%∆r) magnitudes, with a frequency of 0.5 Hz for a duration of 24 h, to recapitulate the mechanical stretches experienced by the cells in vivo. At the end of each experiment, RNA was collected for downstream RNA-Seq and qPCR applications or utilized for imaging by atomic force microscopy. To verify that the experimental system delivered sufficient cyclic stretch, the cells were stained for actin (conjugated phalloidin fluorescein isothiocyanate, Sigma-Aldrich, St. Louis, MO, USA) and DAPI nuclear counterstain following the 24 h stretch period.

### 2.3. RNA Sequencing

Total RNA was extracted from the HUVECs which had undergone both 10% and 20% equibiaxial stretch using an RNEasy Mini Kit (Qiagen, Hilden, Germany). For RNA-seq analysis, the RNA extracts were submitted to the Weill Cornell Medicine Genomic Core Facility. The RNA quality was assessed through RNA integrity numbers (RINs) using an Agilent Technologies 2100 Bioanalyzer. All the RNA samples used in this study had a RIN of 10 out of 10. The RNA library preps were generated and multiplexed using Illumina’s TruSeq RNA Library Preparation Kit v2 (non-stranded and poly-A selection). Ten nanometers of cDNA was used as input for high-throughput sequencing via Illumina’s HiSeq 4000, producing 51 bp paired-end reads. The sequencing reads were de-multiplexed (bcl2fastq v2.17), checked for quality (FastQC v0.11.5), and trimmed/filtered when appropriate (Trimmomatic v0.36). The resultant high-quality reads were mapped (TopHat2 v2.1.0; Bowtie2 v2.2.6) to the transcriptome sequence reference of the UCSC mm10 genome build. The gene counts were quantified using the Python package HTSeq (v0.11.1). The transcript abundance measures (FPKM values) were quantified using Cufflinks (v2.2.1). The gene counts were then analyzed for differential expression using edgeR [27]. Differentially expressed genes were selected across biological replicates with an adjusted *p*-value of <0.05. Counts per million (CPM) values were calculated and utilized to filter out lowly expressed genes. The genes with CPM < 1 were excluded. The selection of differentially expressed genes was performed through pairwise comparisons of 20% stretch vs. control (non-stretch), 20% stretch vs. 10% stretch, and 10% stretch vs. control.

### 2.4. Pathway Analysis

Gene set enrichment analysis (GSEA) was utilized to perform pathway enrichment analysis on the genes that were found to be differentially expressed in each of the pairwise comparisons [28]. Enrichment analysis was performed against the curated gene sets within the Molecular Signatures Database (Broad Institute). Cytoscape version 3.7 was then utilized to map the interaction networks within the enriched gene sets. To create network maps, the Cytoscape plugin GeneMANIA (University of Toronto) was utilized. GeneMANIA employs the guilt-by-association approach to map interactions among related genes [29]. Functional pathway diagrams were then constructed through a combination of the Cytoscape connectivity values and manual literature searches.

### 2.5. Immunofluorescence and Western Blotting

To functionally validate the molecular changes reported by the RNA-seq and pathway analysis, immunofluorescence and Western blotting were performed. The cells were stained for actin (conjugated phalloidin fluorescein isothiocyanate, Sigma-Aldrich, St. Louis, MO, USA), VE-cadherin (goat IgG mouse VE-cadherin, R&D Systems, Minneapolis, MN, USA), and DAPI nuclear counterstain and visualized using a Zeiss Axio Observer Z1. Western blotting was performed with primary Rabbit anti-VEGFR2 (Cell Signaling Technology #2479), Rabbit anti-Akt (Cell Signaling Technology #9272), and Rabbit anti-GAPDH (Cell Signaling Technology #2118) and secondary Goat anti-Rabbit IgG (Li-Cor, Lincoln, NE, USA).

### 2.6. Quantitative PCR

The expression analysis of target genes by quantitative PCR (qPCR) was performed as previously described [30]. The primer sequences are listed in Supplementary Table S1.

Relative gene expression was calculated according to the 2^−ΔΔCt^ method and was normalized to the expression of the housekeeping gene GAPDH. Pairwise comparisons of 20% stretch vs. control and 10% stretch vs. control were performed to determine standard deviation and mean gene expression, in which the stretch samples were normalized to the control. A one-way analysis of variance (ANOVA), followed by a post-hoc Fisher’s LSD multiple comparison test, was utilized to determine statistical significance. A *p*-value < 0.05 was deemed statistically significant.

### 2.7. Atomic Force Microscopy

An MFP-3D-Bio atomic force microscope (Asylum Research, Santa Barbara, CA, USA) was used for all the atomic force microscopy (AFM) measurements. All the AFM measurements were carried out on live cells with the probe immersed in a cell growth medium kept at a temperature of 37 °C using an AFM Petri dish heater (Asylum Research, Santa Barbara, CA, USA). Young’s moduli of live cell monolayers were obtained using a silicon nitride cantilever (Novascan, Ames, IA) with a spherical borosilicate glass probe of 5 μm in diameter. The deflection sensitivity of the cantilever was measured using glass as a stiff surface, while the cantilever spring constant was determined through analysis of the power spectral density (PSD) of the cantilever thermal fluctuations. The AFM software (Asylum Research, Santa Barbara, CA, USA) was utilized to record the PSD and fit the theoretical PSD of a simple harmonic oscillator to the experimental data. The fit described the data accurately, yielding a cantilever stiffness of k = 0.015 N/m, which was consistent with the nominal stiffness (0.02 N/m) reported by the manufacturer. The cell monolayers were positioned under the AFM tip, and the force versus displacement curves were captured on a 32 × 32 grid spanning 80 × 80 μm. Each force curve was recorded at a rate of 4 μm/s and indented at a maximum distance of 200 nm. Low-quality indentation curves (i.e., curves with undefined contact point or non-monotonous slope) were rejected.

The indentation was sufficiently small compared to the height of the cell, allowing the Hertz–Sneddon (HS) model to be used to fit the indentation curves using the AFM software. According to the HS model, the force (denoted as *F*) versus indentation (denoted as *δ*) experienced by a paraboloidal indenter with curvature radius *R* is *F* = 4/3[*E*/(1 − ν^2^)]*R*^1/2^ *δ* 3/2, where *E* and ν denote the cell’s Young’s modulus and Poisson’s ratio, respectively. The data were then fitted from the point of contact between the spherical probe and the sample (chosen to be the point at which the derivative of the force distance curve became nonzero) to the maximum indentation value. The best fit yielded the contact point height and the Young’s modulus of the cell, assuming a cell’s Poisson ratio = 0.45, and the nominal indenter curvature radius *R* = 2.5 μm. The fitted Young’s modulus was utilized to construct the stiffness maps, whereas the contact point height was utilized to map the sample topography. The topography data were used to locate the regions of the stiffness maps where the sample thickness was <1 μm. These regions were occupied by the substrate and the cell periphery and were excluded from the statistical analysis carried out to assess the average stiffness of the cell’s body. The statistical distribution of the AFM measurements was displayed using box plots, in which the horizontal line is representative of the median, the box is representative of the data from the first to third quartile, and the whiskers are representative of the data minimum and maximum. Statistical analyses were conducted using paired t-tests, in which *p* < 0.05 was indicative of statistical significance.

## 3. Results

### 3.1. Cyclic Stretch Validation

**Figure 1:**
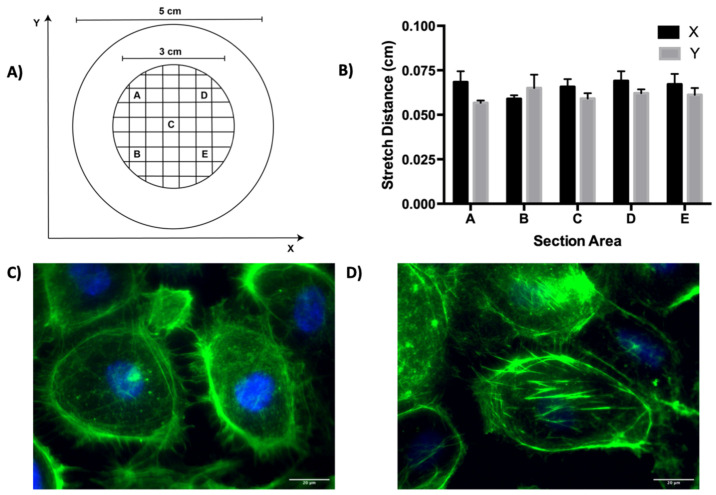
Calibration of the biaxial stretch system. (**A**) Schematic of the sectioned PDMS membrane utilized for calibration. Each box (A–E) is 0.25 cm × 0.25 cm. (**B**) Membrane displacement after stretch at each of the five locations indicated in (**A**). (**C**) Fluorescent image demonstrating staining of actin (green) and DAPI counterstain (blue) after 24 h of static culture on a PDMS membrane at 43× magnification. (**D**) Fluorescent image demonstrating actin staining (green) and DAPI counterstain (blue) after 24 h of biaxial stretch at 43× magnification. Scale bar represents 20 µm.

To validate that the equibiaxial stretch was delivered to the cellular system, a calibration of the cyclic stretch apparatus was performed by sectioning a PDMS membrane into five evenly distributed locations and by determining the mean displacement induced by stretch in the x and y directions across the five technical replicates (Figure 1A). In each of the five locations, there was a mean displacement of 0.0635 cm in both the x and y directions after stretch, indicating that equibiaxial cyclic stretch was imposed on the system (Figure 1B). To verify that the ECs experienced cyclic stretch within the cellular system, F-actin staining was performed on the ECs prior to and after stretch. In the static ECs, the staining is primarily concentrated on the cortical actin, with a limited appearance of actin stress fibers (Figure 1C). However, Figure 1D demonstrates the clear appearance of actin stress fibers and an elongated morphology in the stretched ECs, which is a hallmark of the actin remodeling which occurs in response to cyclic stretch [31].

### 3.2. Cell Cycle Regulation

**Figure 2:**
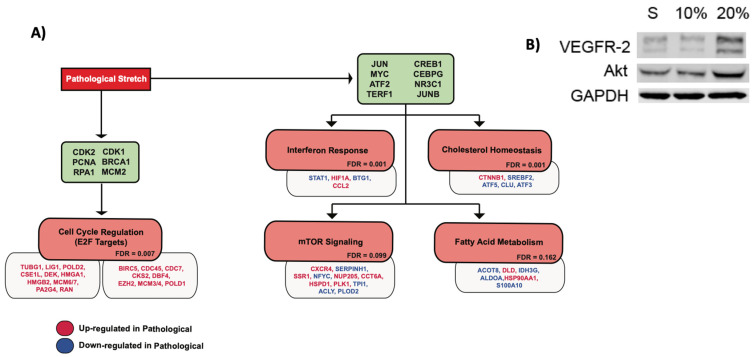
Endothelial response to pathological cyclic stretch. (**A**) Schematic summarizing the endothelial response to pathological magnitudes of cyclic stretch (20%). Green nodes list sets of upstream regulatory genes that correspond to the downstream cellular responses subsequently listed in red nodes. Downstream cellular responses were identified by GSEA analysis. The false discovery rate (FDR) of each identified gene set is listed. An FDR < 0.25 was utilized as the threshold for further investigation. Relevant target genes within each response are listed below the respective node. Target genes colored red are upregulated at 20% stretch while genes colored blue are downregulated at 20% stretch, relative to the static (no stretch) control. (**B**) Western blot analysis of protein modulators involved in the E2F response to pathological cyclic stretch. S represents expression after 24 h of static culture, 10% represents expression after 24 h of 10% cyclic stretch, and 20% represents expression after 24 h of 20% cyclic stretch.

It has previously been reported that there is increased endothelial proliferation under higher magnitudes of cyclic stretch [32]. The GSEA analysis of the 10% sequencing data demonstrated that there is a significant upregulation of the genes associated with cell cycle progression in the 20% stretch relative to the 10% stretch, specifically those downstream of the E2F family of transcription factors (Figure 2A). To validate the proliferative response, Western blot analysis of VEGFR2 and Akt were performed (Figure 2B). The protein expressions of both were upregulated in response to the 20% stretch relative to the 10% stretch and the static conditions.

**Figure 3:**
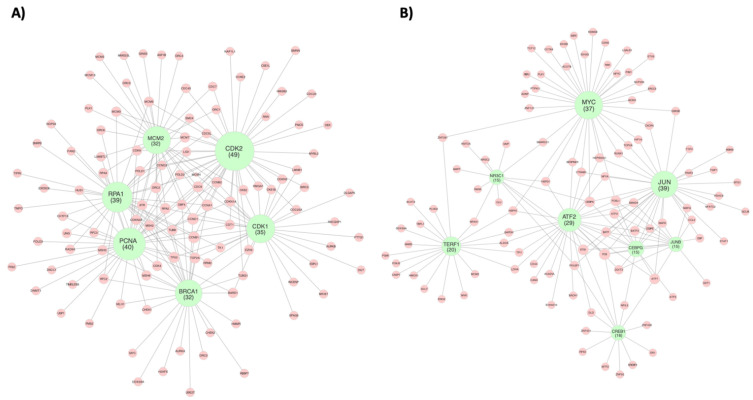
Regulatory gene networks governing the endothelial response to cyclic stretch. Networks represent (**A**) endothelial cell cycle regulation and (**B**) endothelial transcriptional regulation in response to pathological magnitudes of cyclic stretch (20%). Green nodes represent regulatory genes that were identified due to their high number of network connections, which in turn are signified by red nodes representing target genes. Node size of regulatory genes correlates with the number of direct network connections for the respective node. Network was constructed using the GeneMANIA Cytoscape plugin, which utilizes a guilt-by-association approach to map physical interactions between input genes. The input gene list was curated from gene sets identified by GSEA analysis. Only direct first neighbor connections to the regulatory genes are shown here. The full network maps can be seen in Supplementary Figures S1 and S2.

The pathway analysis was performed to delineate the central regulatory network driving the cell cycle progression under the 20% stretch. To do this, the relevant connections between the target genes and the central network nodes were mapped (Figure 3A). Of the E2F target genes, *TUBG1*, *POLD2*, *CSE1L*, *DEK*, *HMGA1*, *HMGB2*, *MCM6/7*, *PA2G4*, *RAN*, *BIRC5*, *CDC45*, *CDC7*, *CKS2*, *DBF4*, *EZH2*, *MCM3/4*, *and POLD1* maintained connections to *CDK2*, which was the central node with the greatest network connectivity (Table 1). *MCM2* had the second greatest network connectivity, maintaining connections to *MCM3/4*, *MCM6/7*, *CDC45*, *CDC7*, *CKS2*, and *DBF4.* MCM2 is a minichromosome maintenance protein that, along with MCM3-7, makes up the protein complex which forms the core of the DNA helicase during DNA replication [33]. The upregulation of the MCM proteins with greater magnitudes of cyclic stretch may provide a molecular basis for the increased rate of DNA synthesis that underlies the cellular response to pathological stretch [34]. The activation of RPA1, a replication protein, and PCNA, a cofactor for DNA polymerase-∂, in response to cyclic stretch may also play a role in this process, as both genes were connected to target genes associated with the enzymes involved in DNA replication (*MCM3/4*, *MCM6/7*, *POLD1*, *POLD2*, and *LIG1*). Although *RPA1* had the third greatest number of connections in the cell cycle connectivity network as a whole (Supplementary Figure S1), *CDK1* had the third most connections to the *E2F* target genes, emphasizing the importance of CDKs in E2F pathway regulation. *CDK1* was connected to *HMGA1*, *BIRC5*, *CKS2*, *EZH2*, and *MCM4*.

### 3.3. Inflammatory Response

Continuous cyclic stretch at high magnitudes may induce the inflammatory secretion of cytokines [35]. Interferons are antiviral factors that are responsible for immune activation through cytokine release [36]. Of the relevant genes associated with interferon response identified by the GSEA analysis, *STAT1*, *HIF1A*, *BTG1*, and *CCL2* were differentially expressed at 20% stretch relative to 10% stretch (Figure 2). The lesser-known *CCL2* is a cytokine and likely modulates inflammatory response, while *BTG1* is an antiproliferative factor which should be considered a result of the inflammatory response. Each of these genes has been implicated in the interferon-mediated inflammatory response [37,38]. Pathway analysis was conducted to identify the relevant connections between the interferon-associated genes and the overall regulatory network. *STAT1*, *HIF1A*, *BTG1*, and *CCL2* each maintained a connection to the transcription factor *JUN*, while *HIF1A* also maintained a connection to the transcription factor *MYC*, and *CCL2* maintained a connection to the transcription factor *JUNB* (Figure 3B). The genes identified as being involved in the cyclic stretch-mediated cholesterol homeostasis in Figure 2, namely *SREBF2*, a sterol regulatory element binding transcription factor, and *ATF3*, an activating transcription factor 3, have also been implicated in the endothelial inflammatory response [39,40]. *SREBF2* was connected to *CREB1*, while *ATF3* was connected *to JUNB*, *JUN*, *ATF2*, and *CEBPG* (Table 2).

### 3.4. Fatty Acid Metabolism

A pathological vascular phenotype has been associated with an increase in glucose metabolism and a decrease in the metabolic utilization of fatty acids [33,41]. The GSEA analysis was utilized to determine the differentially expressed genes associated with the metabolic changes in response to the pathological magnitudes of the cyclic stretch. Of those identified in the gene set, *DLD* and *HSP90AA1* were upregulated, while *ACOT8*, *IDH3G*, *ALDOA*, and *S100A10* were downregulated (Figure 2). *ATF2* and *MYC* were the central regulators of these metabolic genes; *ATF2* maintained connections to *DLD*, *ALDOA*, and *S100A10*, while *MYC* maintained connections to *ACOT8*, *IDH3G*, and *HSP90AA1* (Table 2).

### 3.5. mTOR Signaling

Vascular mTOR signaling has been implicated in both physiological and pathological processes [42]. Using the GSEA analysis, we delineated the mTOR targets which were specifically involved in the pathological mTOR signaling. Of these, *CXCR4*, *SSR1*, *NUP205*, *CCT6A*, *HSPD1*, and *PLK1* were upregulated in the 20% stretch, while *SERPINH1*, *NFYC*, *TPI1*, *ACLY*, and *PLOD2* were downregulated (Figure 2). *MYC* was found to be the primary central regulator of the *mTOR* target genes, maintaining connections to *CXCR4*, *SSR1*, *NUP205*, *CCT6A*, *HSPD1*, *PLK1*, *SERPINH1*, and *NFYC. ATF2* and *TERF1* each had three connections; *ATF2* was connected to *HSPD1*, *SERPINH1*, and *TPI1*, while *TERF1* was connected to *TPI1*, *ACLY*, and *PLOD2*. *JUN* and *NR3C1* each had one connection, to *CXCR4* and *HSPD1*, respectively (Table 2).

### 3.6. Regulation of Cell Stiffness by Cyclic Stretch

**Figure 4:**
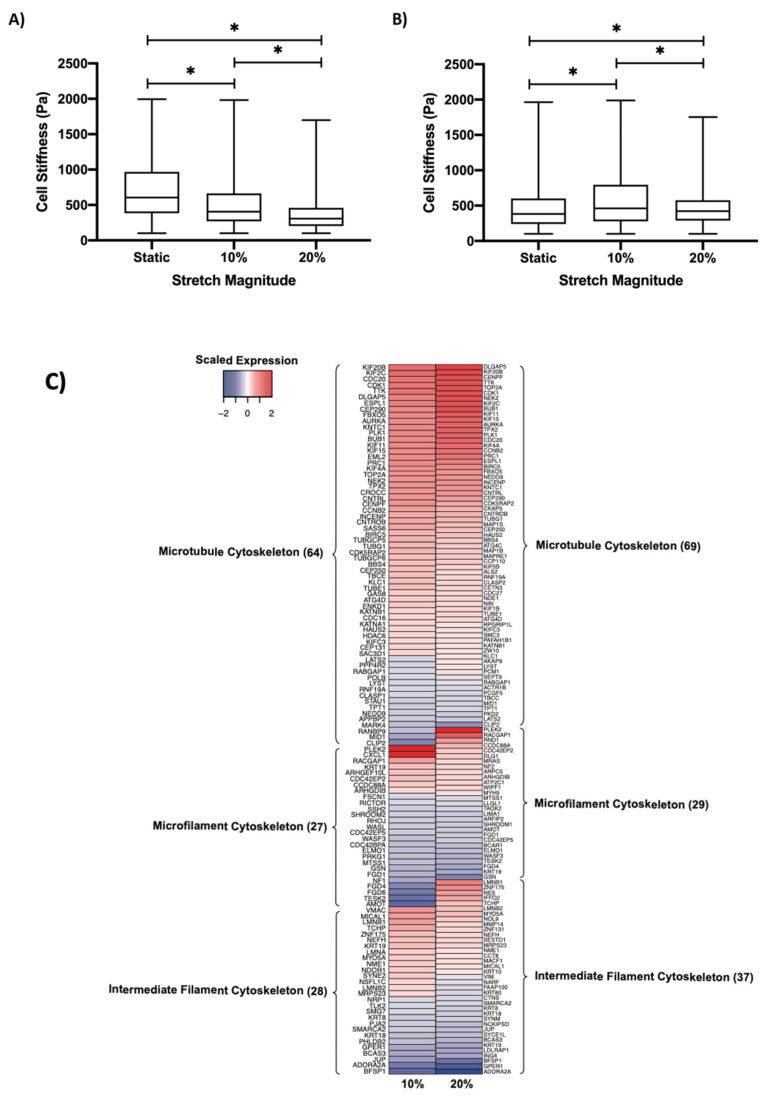

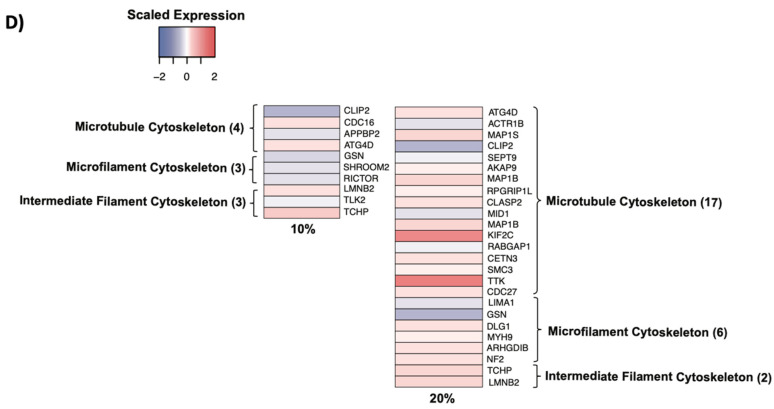
Regulation of the cytoskeleton by cyclic stretch. Cell stiffness of (**A**) HUVECs and (**B**) rapamycin-treated HUVECs as determined through AFM. * indicates *p* < 0.05 between respective groups. (**C**) Heatmap demonstrating the expression of cytoskeletal component genes grouped into their respective classifications in response to 10% and 20% stretch. (**D**) Heatmap demonstrating the expression of cytoskeletal mTOR-target genes grouped into their respective classifications in response to 10% and 20% stretch. For both (**C**,**D**), expression was normalized to the static (no stretch) control. Cytoskeletal genes were identified through the Molecular Signatures Database.

**Figure 5:**
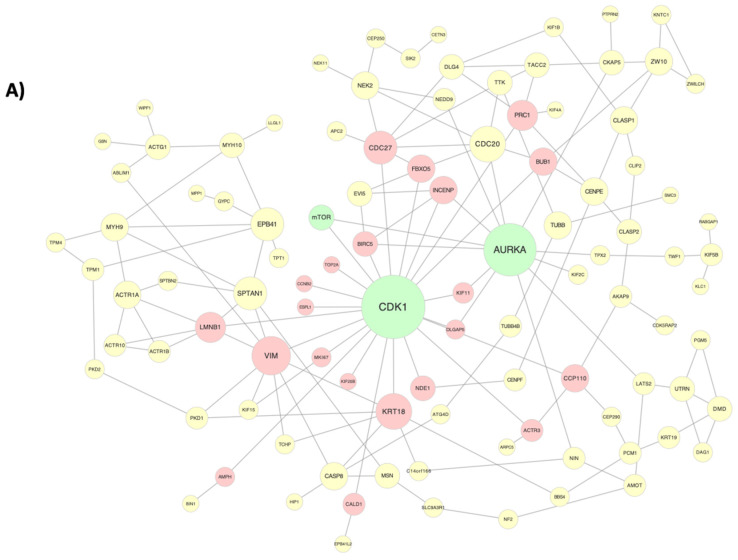

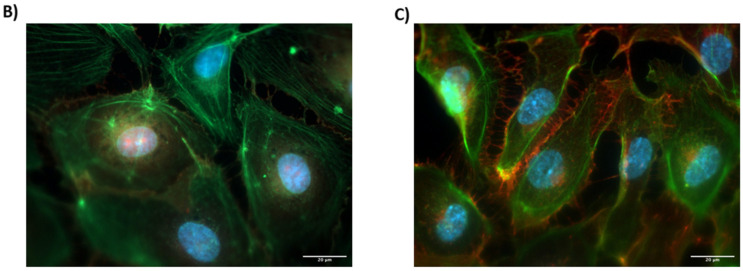
Cytoskeletal activation by cyclic stretch. (**A**) Regulatory network highlighting the connections between cytoskeletal component genes in response to 20% stretch. Green nodes represent central regulators, while red and yellow nodes represent first and second degree connections downstream of CDK1, respectively. (**B**) Fluorescent image demonstrating staining of actin (green), VE-cadherin staining (red), and DAPI counterstain (blue) after 24 h of static culture on a PDMS membrane at 43× magnification. Scale bar represents 20 µm. (**C**) Fluorescent image demonstrating actin staining (green), VE-cadherin staining (red), and DAPI counterstain (blue) after 24 h of biaxial stretch at 43× magnification.

To determine the relationship between cyclic stretch and cell stiffness, we utilized AFM to measure the stiffness of the ECs subjected to 10% and 20% stretch. We found that the cyclic stretch decreased the EC stiffness in a dose-dependent manner, with a median stiffness decreasing from 606 Pa to 405 Pa in response to the 10% stretch and to 306 Pa in response to 20% stretch (Figure 4A). We then treated ECs with rapamycin, a known inhibitor of the mTOR kinase activity, which has previously been demonstrated to play a role in cytoskeletal reorganization, and found a reversal of the previously observed trend, with median stiffness increasing to 461 Pa in response to the 10% stretch and to 421 Pa in response to the 20% stretch (Figure 4B) [43]. The initial decrease in stiffness from 10% to 20% stretch correlated with an increased modulation of the cytoskeletal component genes in response to the 20% stretch (Figure 4C). Of these genes, we mapped the expression of those downstream of *mTOR* and found a greater number of *mTOR* target genes in response to the 20% stretch relative to the 10% stretch (Figure 4D). We then constructed a connectivity network consisting of *mTOR* and the cytoskeletal component genes (Figure 5A). *AURKA* and *CDK1* were identified as central regulators, both of which were connected to *mTOR*.

### 3.7. Cytoskeletal Remodeling

The ECs showed increased localization of VE-cadherin (red) to cell junctions when stretched (Figure 5C) compared to the unstretched controls (Figure 5B). In the unstretched controls, VE-cadherin was preferentially located in the cytoplasm. Differential cell morphology, as highlighted by the actin stain (green), was observed compared with the controls, and it also varied as a function of the distance from the center of the substrate, with greater cell polarity observed further away from center.

### 3.8. Shear Stress Comparison

**Figure 6:**
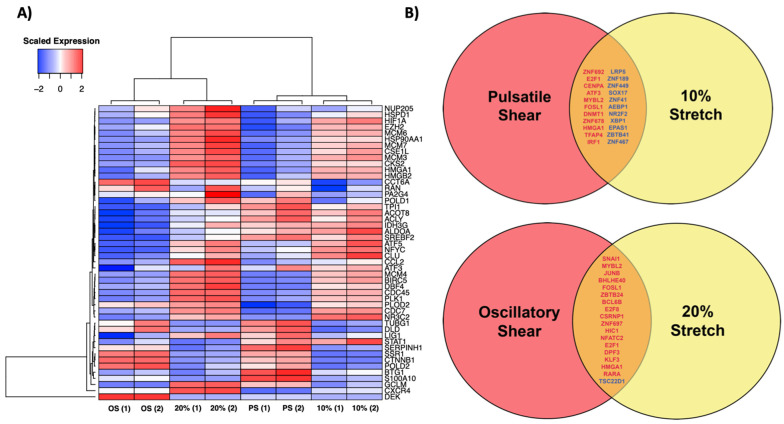
Comparison of gene expression changes in response to shear stress and cyclic stretch. (**A**) Heatmap profiling expression changes of network target genes identified in Figure 3 in response to oscillatory shear, pulsatile shear (PS), 20% stretch, and 10% stretch. OS and PS expression data were compiled from previously published data by Ajami et al., GEO Accession Number GSE103672. For each condition, expression was normalized to the static (no shear/stretch) control and scaled by row. Hierarchical clustering analysis was performed on each column and row. (**B**) Identification of transcription factors that demonstrated changes in expression between both pulsatile shear and 10% stretch and between both oscillatory shear and 20% stretch. Transcription factors colored in red are upregulated in response to the respective conditions, while transcription factors colored in blue are downregulated.

Shear stress and cyclic stretch are complementary hemodynamic forces that act on the endothelium. To determine whether these forces impart similar downstream expressional changes, we compared the expression of the cyclic stretch effector genes identified in Figure 2 in response to the 20% cyclic stretch, the 10% cyclic stretch, the oscillatory shear stress, and the pulsatile shear stress (Figure 6A). Previously published oscillatory and pulsatile shear stress data (GEO Accession Number GSE103672) were reanalyzed for the purposes of this study [8]. These data were obtained from HUVECs exposed to a shear stress of 12 ± 5 dyn/cm2 (pulsatile shear stress) or 0.5 ± 5 dyn/cm2 (oscillatory shear stress). Among the mapped effector genes, the 10% cyclic stretch expression clustered with the pulsatile shear expression, while the 20% cyclic stretch expression clustered with the oscillatory shear expression, suggesting that the 10% cyclic stretch imposes downstream expressional changes similar to those of the pulsatile shear stress, while the 20% cyclic stretch imposes downstream expressional changes to those of the oscillatory shear stress. We then conducted pairwise comparisons to determine whether there are shared transcriptional regulators between each clustered pair (Figure 6B). Seventeen transcription factors were commonly upregulated, and one transcription factor was commonly downregulated between the 20% cyclic stretch and the oscillatory shear stress, while eleven transcription factors were commonly upregulated and downregulated between the 10% cyclic stretch and the pulsatile shear stress.

### 3.9. qPCR Expression Validation

**Figure 7:**
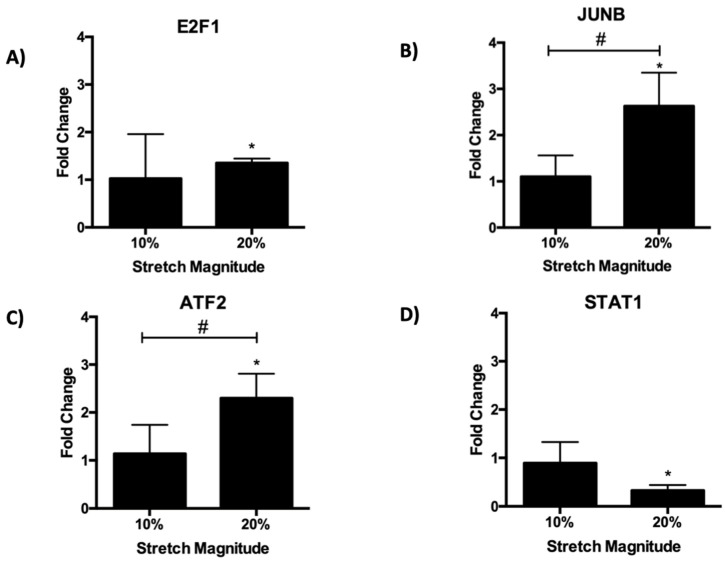
qPCR validation of RNA-seq. Gene expression of (**A**) E2F1, (**B**) JUNB, (**C**) ATF2, and (**D**) STAT1 was determined in response to 24 h of 10% and 20% cyclic stretch. Fold change was calculated by normalizing expression to static controls (fold change = 1). * indicates *p* < 0.05 relative to the static control using one-way ANOVA followed by a post-hoc Fisher’s LSD multiple comparison test, while # indicates *p* < 0.05 between stretch groups.

The expression of four regulatory target genes, *E2F1*, *STAT1*, *JUNB*, and *ATF2* was evaluated by quantitative PCR to verify the RNA-seq dataset. For *E2F1*, *JUNB*, and *ATF2*, there was a significant increase in expression in response to the 20% stretch (Figure 7A–C). Meanwhile, there was a significant decrease in *STAT1* expression in response to the 20% stretch (6D), thus verifying the differential expression observed in the RNA-seq data. The non-significant changes in expression in response to the 10% stretch serve as a further validation, as these target genes were only differentially expressed at the magnitude of the 20% stretch.

## 4. Discussion

This study utilized a systems biology-based approach to map the molecular networks and functional responses that govern the endothelial phenotype induced by the physiological and pathological magnitudes of cyclic stretch. Previous studies have investigated the effects of cyclic stretch by using experimental systems that primarily deliver uniaxial stretch at set magnitudes [21]. However, these studies provide limited insight into the differential endothelial responses to the increasing magnitudes of equibiaxial cyclic stretch. This study utilized RNA-seq data collected from ECs subjected to 10% and 20% equibiaxial cyclic stretch to conduct functional pathway analyses and to visualize the gene regulatory networks, providing comprehensive insights into the temporal relationships between endothelial gene expression and the relevant phenotypic responses.

Low-magnitude cyclic stretch (5–10% ∆r) has largely been considered a physiological stretch, associated with an atheroprotective phenotype, while high-magnitude cyclic stretch (>20% ∆r) has largely been considered a pathological stretch, associated with an atheroprone phenotype [24]. The pathological stretch coupled with oscillatory shear stress, imparted by disturbed blood flow, has been implicated in the pathogenesis of vascular disease [44,45,46]. Thus, the aim of this study was twofold. First, we aimed to determine the molecular signatures which constitute the endothelial state during the pathological stretch. Second, we aimed to compare these molecular signatures to the molecular signatures that govern the endothelial state during oscillatory shear stress.

### 4.1. Endothelial Response to Cyclic Stretch

Following overexpression analysis, we identified four key endothelial responses to cyclic stretch: cell cycle regulation, interferon response, fatty acid metabolism, and mTOR signaling. Cell cycle hyperactivity has been implicated in the pathological progression of vascular disease [47]. In this study, we identified a cohort of cell cycle-related genes, all downstream targets of the *E2F* family of the transcription factors that are involved in driving the progression of the cell cycle under the pathological cyclic stretch.

Outside of their role in the cell cycle, the E2F transcription factors have also been implicated in the hydrogen peroxide (H2O2)-induced downregulation of the tetrahydrobiopterin salvage enzyme DHFR (dihydrofolate reductase), causing eNOS (endothelial NO synthase) uncoupling and a subsequent increase in blood pressure [48]. Because of this dual role, the targeting of the E2F transcription factors may be a viable therapeutic option to mitigate the progression of vascular disease. The role of hyperactive cell cycle activity and proliferation in the pathological progression of vascular disease was confirmed by protein expression analysis as the expression of both VEGFR2 and Akt, which are known markers of endothelial proliferation modulated by E2F activity, increased in response to the pathological magnitudes of cyclic stretch [49,50].

The genes identified in the interferon response (*STAT1*, *HIF1A*, *BTG1*, *CCL2*) and cholesterol homeostasis (*ATF3*, *SREBF2*) have previously been implicated in the inflammatory response, which occurs during the onset of vascular disease [37,38,39,40,51]. *ATF3* and *SREBF2* have both been demonstrated to mediate the effects of high-density lipoprotein (HDL) and low-density lipoprotein (LDL), respectively [40,52]. The downregulation of *ATF3* in response to pathological stretch may suggest an impairment of the ATF3-dependent atheroprotective effects of HDL during the onset of vascular disease [53]. The development of primary aldosteronism is another factor contributing to vascular disease progression and is the most common cause of reversible vascular disease [54]. Overactive mTOR signaling has been demonstrated to contribute to primary aldosteronism; mTORC1 complex inhibition has been associated with decreased levels of plasma aldosterone and decreased blood pressure [55,56]. Thus, the upregulation of mTOR pathway components in response to pathological stretch may represent another underlying molecular contributor driving vascular disease.

Vascular disease is also characterized by the preferential utilization of glucose for metabolism rather than fatty acids [33,41]. We found that this metabolic profile may be due in part to the upregulation of *DLD* and the downregulation of *ACOT8* in response to pathological stretch. *DLD* forms a subunit in the pyruvate dehydrogenase complex, a highly regulated enzyme preceding the tricarboxylic acid cycle during glucose metabolism [57]. Meanwhile, *ACOT8* plays a role in ß-oxidation during the metabolism of fatty acids [58].

Each of the identified functional responses was driven by a central regulatory network. This regulatory network consisted of a cohort of transcription factors (*JUN*, *MYC*, *ATF2*, *TERF1*, *CREB1*, *CEBPG*, *NR3C1*, and *JUNB*) that were specifically upregulated in response to the pathological cyclic stretch relative to the physiological cyclic stretch. Of these functional responses, we found that the inflammatory response was mediated by *JUN*, *MYC*, and *JUNB*; cholesterol homeostasis was mediated by *CREB1*, *JUNB*, *JUN*, *ATF2*, and *CEBPG*; fatty acid metabolism was mediated by *ATF2* and *MYC*; and mTOR signaling was mediated by *MYC*, *ATF2*, *TERF1*, *JUN*, and *NR3C1*.

The combined activity of these transcription factors may play an Important role in coordinating the molecular transition that occurs as ECs shift from an atheroprotective to an atheroprone phenotype in response to the increasingly pathological magnitudes of cyclic stretch, culminating in vascular disease.

### 4.2. Effect of Cyclic Stretch on the Endothelial Cytoskeleton

The endothelial cytoskeleton has been shown to be involved in vascular disease [59]. We aimed to evaluate whether cyclic stretch impacted the EC cytoskeletal reorganization by determining cell stiffness in response to physiological and pathological cyclic stretch. We found that the cytoskeletal response is dose-dependent and that the ECs become softer at higher magnitudes of cyclic stretch. Thus, a decrease in EC stiffness may contribute to the development of an atheroprone phenotype. This decrease in stiffness was reversed by the treatment with rapamycin, a known inhibitor of mTOR [60]. We found that there was a greater number of differentially expressed cytoskeletal component genes in response to the 20% stretch relative than to the 10% stretch, signifying that the 20% stretch leads to an increase in the regulation of microtubule, microfilament, and intermediate filament genes. This provides a molecular basis for the larger stiffness decrease that was observed, given that alternations in cell stiffness depend on the corresponding alterations in the cytoskeleton [61]. There were also a greater number of mTOR target cytoskeletal genes that were differentially expressed in the 20% stretch, potentially explaining why the rapamycin treatment had a more significant impact on the stiffness in response to the 20% stretch rather than the 10% stretch. Furthermore, the VE-cadherin mobilization in response to stretch suggests a highly involved cellular response, with a notable example in the fact that VEGFR2 is known to associate with VE-cadherin near cell membranes in a process that modulates receptor functionality [62].

To establish a mechanism for stiffness regulation by cyclic stretch, we constructed a connectivity network consisting of differentially expressed cytoskeletal component genes and mTOR. Two central regulators were identified, *AURKA* and *CDK1*, which were both connected to *mTOR*. *AURKA* has been shown to drive mTOR activation, while *CDK1* has previously been demonstrated to help modulate downstream mTOR effectors [63,64]. Both of these proteins are regulators of microtubule behavior [45,65]. Thus, we hypothesize a potential regulatory mechanism in which *AURKA* is stimulated by an upstream mechanosensitive pathway to cyclic stretch and leads to downstream mTOR activation. *CDK1*, which was connected to an additional 21 downstream cytoskeletal component genes (*CDC27*, *PRC1*, *BUB1*, *FBXO5*, *INCENP*, *BIRC5*, *KIF11*, *DLGAP5*, *TOP2A*, *CCNB2*, *ESPL1*, *MKI67*, *KIF20B*, *NDE1*, *KRT18*, *LMNB1*, *VIM*, *AMPH*, *CALD1*, *CCP110*, and *ACTR3*) may then regulate the downstream effects of mTOR kinase activity on the cytoskeleton. The activation of this mechanosensitive pathway is likely to be magnitude-dependent, with a greater magnitude of stretch leading to greater pathway activation, which would explain the increased number of differentially expressed mTOR target genes found in response to the 20% stretch.

### 4.3. Comparison of Endothelial Response to Cyclic Stretch and Shear Stress

Because of the complementary nature of cyclic stretch and shear stress as hemodynamic forces, we wanted to determine whether similar molecular networks regulate the endothelial response to each. We compared the previously published data that mapped the molecular response of ECs to pulsatile and oscillatory shear stress, as being representative of atheroprotective and atheroprone conditions, respectively [8]. After mapping the expression of the identified effector genes, we found that the expressional pattern of ECs in response to the 10% stretch was most closely related to that of the ECs in response to pulsatile shear stress, while the expressional pattern in response to the 20% stretch was most closely related to that of the ECs in response to oscillatory shear stress. This suggests that the physiological and pathological magnitudes of cyclic stretch and shear stress impart similar molecular and, in turn, functional responses to the endothelium to coordinate an atheroprotective and atheroprone phenotype, respectively.

To determine whether common transcriptional regulators exist in the responses to cyclic stretch and shear stress, we identified transcription factors that were differentially expressed in response to both the 10% stretch and pulsatile shear stress and the 20% stretch and oscillatory shear stress, respectively. We found 22 transcription factors that were commonly expressed between the 10% stretch and the pulsatile shear stress, 11 of which were upregulated (*ZNF692*, *E2F1*, *CENPA*, *ATF3*, *MYBL2*, *FOSL1*, *DNMT1*, *ZNF678*, *HMGA1*, *TFAP4*, and *IRF1*) and 11 of which were downregulated *(LRP5*, *ZNF189*, *ZNF449*, *SOX17*, *ZNF41*, *AEBP1*, *NR2F2*, *XBP1*, *EPAS1*, *ZBTB41*, and *ZNF467*). In addition, we found 17 transcription factors commonly upregulated between the 20% stretch and the oscillatory shear stress (*SNAI1*, *MYBL2*, *JUNB*, *BHLHE40*, *FOSL1*, *ZBTB24*, *BCL6B*, *E2F8*, *CSRNP1*, *ZNF697*, *HIC1*, *NFATC2*, *E2F1*, *DPF3*, *KLF3*, *HMGA1*, and *RARA*) and 1 transcription factor that was commonly downregulated (*TSC22D1*). The common upregulation of *SNAI1* in response to both the 20% stretch and the oscillatory shear stress is particularly of interest, given its central role as a transcriptional regulator of the endothelial to mesenchymal transition (EndMT) and the increased speculation surrounding the role of EndMT in vascular disease progression [66]. Although our analysis is preliminary in scope, the transcription factors provided here provide a basis for future investigations into the shared regulatory networks that drive the molecular response to cyclic stretch and shear stress. Specifically, future genomic analyses evaluating the potential synergistic effects of pathological cyclic stretch and pathological shear stress should be conducted using in vitro systems capable of delivering concurrent cyclic stretch and shear stress, as previously established [67].

## 5. Conclusions

In this study, we present a thorough overview of the functional and molecular endothelial responses to pathological and physiological cyclic stretch. Through detailed network analysis, we delineated the regulatory transcriptional networks driving the progression from an atheroprotective to an atheroprone endothelial phenotype induced by equibiaxial cyclic stretch. The genes identified here provide a novel insight as an invaluable predicative framework for the dissection of future clinical data on cohorts of patients with vascular disease; a few of these (S100A10, FOS) have already been identified in clinical genomic analyses of vascular disease [68]. Thus, this study verifies and expands upon the regulatory mechanisms which are currently understood and provides gene targets that lay the foundation for further investigation into the pathogenesis of vascular disease.

## Figures and Tables

**Table 1:** Cell cycle regulatory network interaction list. Connectivity value is included in parentheses next to each regulatory gene and represents the number of node connections associated with each regulatory gene.

CDK2 (49)	PCNA (40)	RPA1 (39)	CDK1 (35)	BRCA1 (32)	MCM2 (32)
PA2G4	USP1	TIPIN	TIPIN	ORC2	RPA3
PAICS	TIMELESS	EXOSC8	EXOSC8	MSH6	ORC2
MYBL2	POLD3	SMC4	SMC4	CCNB1	PLK1
HMGA1	POLD2	TMPO	TMPO	H2AFX	CDK6
CCNE2	POLD1	CHTF18	CHTF18	HMMR	ORC5
HMGB2	RPA1	UNG	UNG	CDK4	L3MBTL1
CDKN3	RPA2	NOP56	NOP56	TUBB	MCM7
CKS2	RPA3	RFC2	RFC2	EZH2	ORC4
CCNA1	RFC2	RFC3	RFC3	MYC	MCM5
CDC25A	RFC3	SNRPB	SNRPB	UBE2T	GINS3
GMNN	CHTF18	RPA3	RPA3	MLH1	CDC6
CKS1B	HUS1	PAN2	PAN2	DBF4	CDK4
CDC20	MLH1	RPA2	RPA2	MSH3	ORC6
CDC7	DSCC1	POLD1	POLD1	CHEK2	MCM3
DBF4	POLE	PCNA	PCNA	RBBP7	DBF4
MCM4	UNG	TOP2A	TOP2A	DDX39A	CCNB2
MCM6	PMS2	MSH3	MSH3	ORC3	CDC45
CDC45	TOP2A	HUS1	HUS1	BARD1	MCM4
ORC2	MSH3	RAD9A	RAD9A	CDKN2A	CKS2
MCM7	MSH6	MSH6	MSH6	TOP2A	CDKN2A
ORC1	RAD9A	MSH2	MSH2	RPA1	RPA1
CDC6	DNMT1	ATR	ATR	TP53	ASF1B
CDT1	MSH2	BRCA1	BRCA1	TUBG1	MCM10
CDKN1A	CHEK1	TK1	TK1	MSH2	RPA2
CCNB1	BARD1	CDK1	CDK1	ATR	ATR
CDK1	CDK6	CCNB1	CCNB1	CHEK1	ORC1
TUBG1	LIG1	CCNA1	CCNA1	CDK1	CCNA1
LMNB1	CDKN2A	ORC6	ORC6	CCNA1	CDC7
CCNB2	TP53	MCM4	MCM4	CCND1	MMS22L
CCND1	CDK4	MCM6	MCM6	AURKA	CCND1
MCM2	CCND3	MCM7	MCM7	CDK2	MCM6
MCM3	CCND1	MCM2	MCM2	RFC2	CDK2
POLD1 POLD2	CDK2 CDC6	MCM3 ORC2	MCM3 ORC2		
RPA1	CDKN1A	CDK2	CDK2		
PCNA	CCNB2	CDC5L	CDC5L		
BRCA1	CDK1	TP53	TP53		
TUBB CDC5L	CCNB1 CDT1	TUBB RRM2	TUBB RRM2		
BIRC5	L3MBTL1				
CCND3					
CDK6					
MSH2 SMC4					

**Table 2:** Transcriptional regulatory network interaction list. Connectivity value is included in parentheses next to each regulatory gene and represents the number of node connections associated with each regulatory gene.

JUN (39)	MYC (37)	TERF1 (20)	ATF2 (29)	NR3C1 (15)	JUNB (15)	CREB1 (16)	CEBPG (15)
	JUN	WARS	HSP90AA1	NR4A1	SAT1	SREBF2	CEBPE
DDIT3	HIF1A	PGMI	FOSL1	MVP	DDIT3	ZNF451	DDIT3
TRAF2 CCL2	HSP90AA1	FOXJ3	CEBPA	CLU	ATF3	JUN	ATF2
	FOSL1	MCM2	BATF3	SMARCC1	CCL2	MYC	ATF7
ATF3 TGIF1 JUNB	CEBPA	HMOX1	JUN	CREB1	SMAD4	NR3C1	BATF
	CTNNB1	TBPL1	ATF7	POU2F1	JUN	ATF7	BATF3
	GSK3B	DDX39A	FOS	CEBPA	BATF	DR1	CEBPA
DBP BATF MAFG	RUNX1	ENO2	CTNNB1	FOS	MAFG	DLD	ATF3
	SERPINH1	ACAT2	CEBPG	JUN	BATF3	ETS1	ATF5
	TCF12	MVK	ATF3	MAFF	MAFK	FOS	MAFK
BATF3 MAFK HIF1A	KDM5B	ZNF281	ETS1	ETS1	ATF2	NFIL3	DBP
	HSPD1	LDHA	DDIT3	KMT2A	ATF7	POU2F1	FOS
	NMI	GAPDH	BACH1	RARA	FOSL1	MTF2	FOSL1
HDAC9 CEBPE NFATC2	CREB1	TPI1	BATF	NR3C2	FOS	RFX3	NFIL3
	CDK6	HSPH1	JUNB	HSPD1	ETS1	ZNF436	JUN
SMAD4 FOS ETS1	CXCR4	NR4A1	SERPINH1			ZNF92	
	TOP2A	PLOD2	SMAD4				
	NFYC	CASP7					
ATF7 ATF2 FOSL1	SMARCC1	ACLY	CANX				
	HSPH1	ALDOA	CD44				
	PIM1		NFYA				
	ZNF281						
	CCT6A		HSPH1				
HSP90AA1 CEBPA CEBPG	ETV3		DLD				
	IDH3B		HSPD1				
CTNNB1 GSK3B RUNX1	GCDH		ALDOA				
	ACOT8		TPI1				
IKBKB NR3C1 STAT1	ZNF121		LDHA				
	NUP205		GAPDH				
POU2F1 CXCR4 TOP2A	PTPN11		S100A10				
	IDH3G		ACADVL				
	SSR1						
	LGALS1						
NFYA MYC CREB1	ERCC3						
	PLK1						
GCLM TCF4 BTG1	ADNP						
	RBPJ						

## Data Availability

Not applicable.

## Supplementary Materials

The following supporting information can be downloaded at: https://www.mdpi.com/article/10.3390/biom12121837/s1.
