# Supplementary material for: Systems Biology Analysis of Temporal Dynamics That Govern Endothelial Response to Cyclic Stretch
Source: Biomolecules. 2022 Dec 8;12(12):1837. doi: 10.3390/biom12121837 (PMC9775567; doi:10.3390/biom12121837)

Supplementary Table S1

Gene	Forward Sequence	Reverse Sequence
**E2F1**	5’-GGATTTCACACCTTTTCCTGGAT-3’	5’-CCTGGAAACTGACCATCAGTACCT-3’
**STAT1**	5’-CTAGTGGAGTGGAAGCGGAG-3’	5’-CACCACAAACGAGCTCTGAA-3’
**JUNB**	5’-GTCACCGAGGAGCAGGAGG-3’	5’-TCTTGTGCAGATCGTCCAGG-3’
**ATF2**	5’-ACCATGGTGCCTAGTGTTCC-3’	5’-GTGGCTGGCTGTTGTAATGA-3’

Supplementary Figure S1

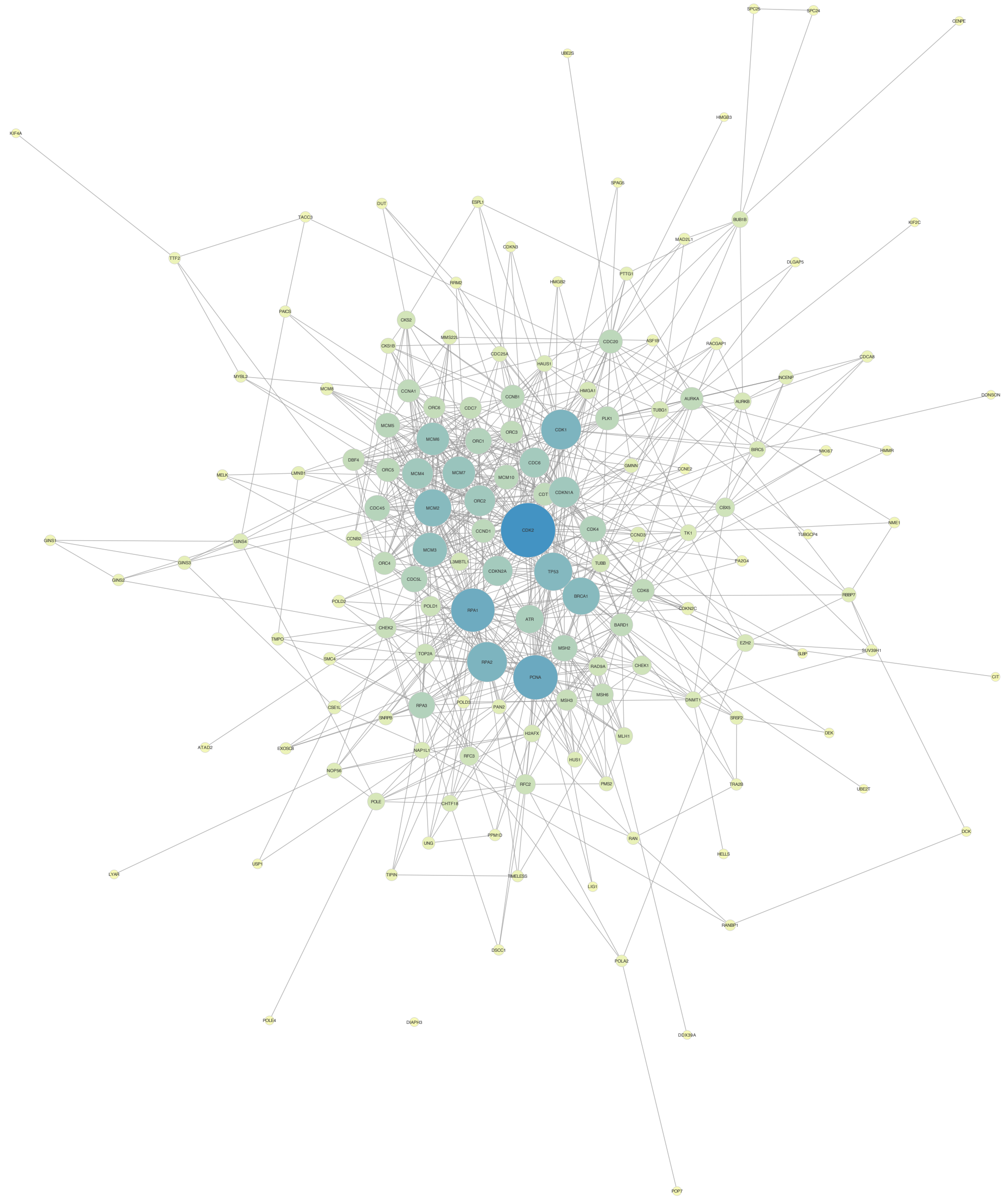

Supplementary Figure S2

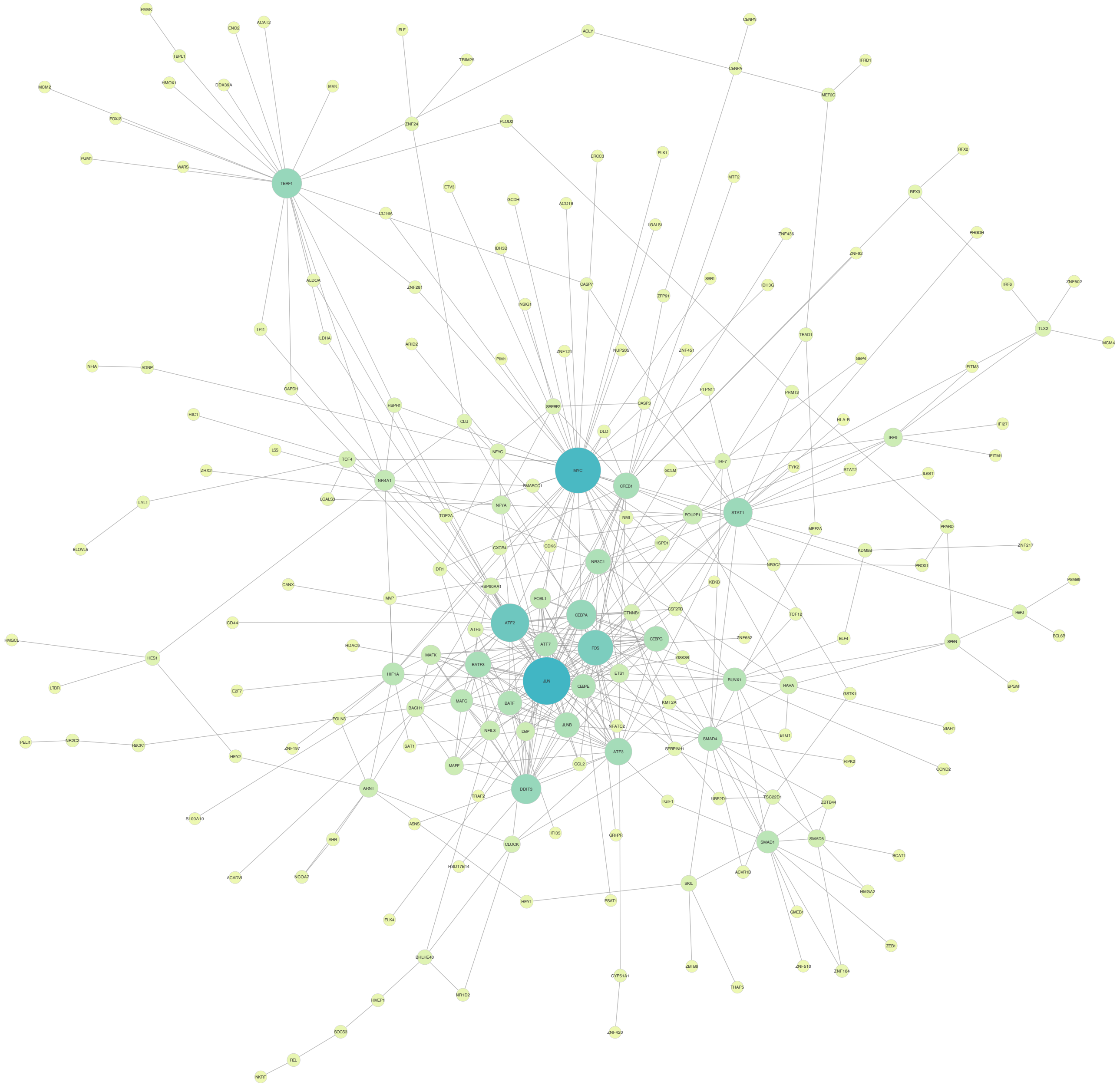

Supplement: Supplementary file 1 [file biomolecules-12-01837-s001.zip › biomolecules-2015358 Supplementary Materials.pdf]
